# Characteristics and Prognosis of Early‐Onset vs. Late‐Onset Colon Cancer: A Propensity Score Matching Analysis Based on Histology

**DOI:** 10.1002/cam4.71681

**Published:** 2026-03-03

**Authors:** Xiaobin Cheng, Tao Xiang, Saisai Wang, Jinhai Wang

**Affiliations:** ^1^ Department of Colorectal Surgery The First Affiliated Hospital, Zhejiang University School of Medicine Hangzhou People's Republic of China; ^2^ Department of Gastrointestinal Surgery The Second Affiliated Hospital of Nanchang University Nanchang People's Republic of China

**Keywords:** colon cancer, histology, prognosis, propensity score matching, SEER database

## Abstract

**Objectives:**

The prognostic value of age at diagnosis in colon cancer is poorly understood. This study evaluated the influence of age at diagnosis and histologic subtype on colon cancer prognosis using data from 180,804 patients in the SEER database.

**Methods:**

Patients were stratified into early‐onset and late‐onset groups and by histology—adenocarcinoma or mucinous adenocarcinoma. Early‐onset colon cancer was associated with more advanced tumor stages. Propensity score matching balanced cohorts for Kaplan–Meier and Cox regression analyses of cancer‐specific survival (CSS) and surgery‐specific survival (SSS).

**Results:**

Survival analyses revealed that patients with early‐onset adenocarcinoma had the most favorable CSS and SSS, whereas those with late‐onset mucinous adenocarcinoma experienced the worst outcomes; no significant age‐related differences emerged histology‐stratified. Importantly, among nonmetastatic patients, early‐onset adenocarcinoma showed significantly poorer SSS compared to late‐onset adenocarcinoma.

**Conclusion:**

These findings emphasize the necessity of integrating age and histologic type for accurate prognostic assessment and personalized treatment strategies in colon cancer.

## 1 Introduction

Colorectal cancer is among the most common and lethal malignancies, and its incidence is increasing among younger individuals. Early‐onset colorectal cancer (EOCRC) is projected to account for 11% of all colon cancers and 23% of all rectal cancers by 2030 [1, 2]. Based on age at diagnosis, colorectal cancer can be categorized into two groups: Early‐onset (i.e., diagnosed before the age of 50) and late‐onset (i.e., diagnosed at age 50 or older) [3]. Colorectal cancer research has undergone a paradigm shift that has increased the focus on the clinical presentation, pathological features, molecular profiles, and survival outcomes of younger patients and shifted it away from their older counterparts [4]. Studies comparing early‐onset and late‐onset colorectal (LOCRC) cancer have indicated that early‐onset cases typically present with an advanced tumor stage and unfavorable features, such as poor differentiation and mucinous or signet‐ring subtypes [5]. Despite the aggressive behavior of early‐onset colorectal cancer, both cohorts have comparable survival outcomes [5, 6]. The better tolerance of younger patients to treatment‐related toxicity may mitigate the negative impact of aggressive tumor biology [4]. However, certain studies suggest that disease‐free survival (DFS) can differ between young patients and older patients [7, 8]. Tumor heterogeneity and differences in covariates may explain the differences in diagnosis between these cohorts.

Rectal cancer and colon cancer exhibit significant differences in tumor biology. Unlike previous studies, the present study focused exclusively on colon cancer. To better clarify the effects of age at onset on tumor biology and prognosis, data on adenocarcinoma and mucinous carcinoma (which are the two main subtypes of colon cancer) were extracted from the Surveillance, Epidemiology, and End Results (SEER) database. The clinicopathological characteristics of patients with adenocarcinoma and those with mucinous carcinoma were compared between patients with early‐ and late‐onset disease. Propensity score matching (PSM) was employed to eliminate confounding bias from observational cohorts before outcome analyses among early‐ and late‐onset patients.

## 2 Methods

### 2.1 Data Source

Data for this study were obtained from the SEER database, which is publicly available and reliable [9]. The SEER registries include comprehensive information on patient demographics, tumor pathology, treatment, and follow‐up details regarding vital status and causes of death. The researchers obtained permission to download the research data files from the SEER database, and no further informed consent was needed.

### 2.2 Patient Selection

Patients diagnosed with colon cancer between 2000 and 2022 (17 registries, November 2022) were identified and selected via SEER*Stat software (version 8.4.3) based on the following inclusion criteria:

1. The International Classification of Diseases for Oncology, Third Edition (ICD‐O‐3) biology codes indicated malignant tumors.
2. ICD‐O‐3 histology codes: 8140/3, 8480/3, 8481/3.
3. ICD‐O‐3 site codes: Colon excluding rectum.

Patients diagnosed during autopsy or on the death certificate were excluded from this study.

The independent variables for this study included demographic characteristics (age, sex, race, marital status, median household income, and rural–urban continuum code residence districts), tumor features (location, histology, grade, and stage), treatment approach (surgery, radiation, and chemotherapy), and survival information (vital status, survival months, survival months flag, and causes of death classification).

### 2.3 Parameters

The cutoff for age at onset was defined as 50 years. ICD‐O‐3 histology code 8140/3 referred to adenocarcinoma, whereas codes 8480/3 and 8481/3 referred to mucinous adenocarcinoma. Patients were categorized based on histology and age at onset into an early‐onset colon cancer (EOCC) group, which included early‐onset adenocarcinoma (EOAC) and early‐onset mucinous adenocarcinoma (EOMC), and a late‐onset colon cancer (LOCC) group, which included late‐onset adenocarcinoma (LOAC) and late‐onset mucinous adenocarcinoma (LOMC). Furthermore, some variables were recategorized and redefined as follows:

- Race was categorized as White, Black, and Other (including American Indian/Alaskan Native and Asian or Pacific Islander).
- Marital status was categorized as married or unmarried (including divorced, separated, single, unmarried or domestic partner, and widowed).
- Location was categorized as left (including the splenic flexure, descending colon, and sigmoid colon) or right (including the cecum, ascending colon, hepatic flexure, and transverse colon).
- Median household income was categorized into four groups: < $55,000, < $55,000, < $65,000, and ≥ $75,000.
- Tumor differentiation was categorized as well differentiated (Grade I/II) or poorly differentiated (Grade III/IV).
- Rural–urban distribution was categorized as metropolitan (counties in metropolitan areas with populations ≥ 1 million), intermediate (counties in metropolitan areas of 250,000 to 1 million and counties in metropolitan areas with populations < 250,000), or nonmetropolitan (counties not adjacent to a metropolitan area).
- Cancer‐specific survival (CSS) and surgery‐specific survival (SSS) were classified as follows: CSS was defined as the time from diagnosis to death attributable to colon cancer, with censoring for living patients or deaths from other causes, based on SEER cause‐specific death classification (“alive or dead of other cause” for censoring; “dead (attributable to this cancer)” for events) and COD (cause of death) to site rec KM (“alive” for censoring; “colon excluding rectum” for events). SSS was defined as CSS restricted to patients who underwent surgery but never received adjuvant therapy.

To minimize the impact of aging and treatment delays on survival outcomes, patients had to meet the following criteria before prognostic analysis [1]: age at onset was no more than 75 years; and [2] the time from diagnosis to treatment was no longer than 2 months. The follow‐up data had to meet the following criteria [1]: vital status was either alive or dead because of colon cancer [2]; the survival months flag was limited to “complete dates are available and there are more than 0 days of survival” or “incomplete dates are available and there cannot be zero days of follow‐up”’; and [3] complete data on tumor grade and stage were available.

### 2.4 Statistical Analysis

Categorical variables were compared using the chi‐square test to assess baseline differences. To minimize confounding and balance cohorts, propensity score matching (PSM) was performed via logistic regression, incorporating sex, race, marital status, primary location, tumor differentiation, tumor stage, median household income, and rural–urban distribution. The nearest‐neighbor algorithm was applied with a 1:4 matching ratio and a caliper of 0.1; standardized mean differences (SMDs) below 0.1 confirmed adequate balance per the PSM guidelines. Kaplan–Meier curves illustrate CSS and SSS, with log‐rank tests for group comparisons. Univariate Cox proportional hazards regression was used to identify prognostic factors associated with CSS and SSS. Significant variables were included in multivariate Cox models, where hazard ratios (HRs) and 95% confidence intervals (CIs) were calculated. Schoenfeld residuals were used to test the proportional hazards assumption (PHA); for violations, we used piecewise Cox models (to capture time‐varying effects via data‐driven cutoff points) and stratified Cox models (to allow separate baseline hazards for nonproportional variables), ensuring PHA compliance and robust inference. All analyses were conducted in R version 4.2.1 (R Foundation for Statistical Computing, Vienna, Austria), with *p* < 0.05 indicating statistical significance.

## 3 Results

### 3.1 Patient Characteristics

A total of 180,804 colon cancer patients were ultimately selected from the SEER database, including 165,085 (91.3%) with adenocarcinoma and 15,719 (8.7%) with mucinous adenocarcinoma. Among these patients, 49.7% were female, and 50.3% were male. Across all histologic types, mucinous adenocarcinoma was predominant in females (51.7%), whereas adenocarcinoma was predominant in males (50.5%) (Supplementary Table S1). Patients were classified into four custom groups on the basis of histology and age at onset: EOAC (*n* = 15,884, 8.8%), LOAC (*n* = 149,201, 82.5%), EOMC (*n* = 1,518, 0.8%), and LOMC (*n* = 14,201, 7.9%). Patients in both the EOCC and LOCC groups were predominantly male. Specifically, the male predominance in EOCC was due mainly to the EOMC subgroup (59.4%). Unlike the other cohorts, the LOMC group exhibited a female predominance (52.9%). The distribution of age at onset varied among different races. EOCC was more common among black patients and patients of other races, whereas LOCC was more common among white patients (*p* < 0.001). Although the distribution of marital status varied to some extent, being married remained the most common marital status across all patient cohorts. The distributions of median household income and rural–urban distribution in the EOCC and LOCC groups were similar, although the P value indicated a significant difference (see Table 1 for details).

**TABLE 1:** Baseline Demographic and Clinical Characteristics of Included Colon Cancer Patients.

Characteristics	EOAC	LOAC	EOMC	LOMC	Overall	*P*
	(*N* = 15,884)	(*N* = 149,201)	(*N* = 1,518)	(*N* = 14,201)	(*N* = 180,804)	
Sex						
Female	7,783 (49.0%)	73,909 (49.5%)	616 (40.6%)	7,518 (52.9%)	89,826 (49.7%)	< 0.001
Male	8,101 (51.0%)	75,292 (50.5%)	902 (59.4%)	6,683 (47.1%)	90,978 (50.3%)	
Race						
Black	2,472 (15.6%)	17,920 (12.0%)	235 (15.5%)	1,459 (10.3%)	22,086 (12.2%)	< 0.001
Others	1,939 (12.2%)	14,257 (9.6%)	155 (10.2%)	992 (7.0%)	17,343 (9.6%)	
White	11,278 (71.0%)	115,870 (77.7%)	1,111 (73.2%)	11,694 (82.3%)	139,953 (77.4%)	
Missing	195 (1.2%)	1,154 (0.8%)	17 (1.1%)	56 (0.4%)	1,422 (0.8%)	
Marital status						
Married	8,553 (53.8%)	73,848 (49.5%)	776 (51.1%)	6,924 (48.8%)	90,101 (49.8%)	< 0.001
Unmarried	6,489 (40.9%)	65,978 (44.2%)	659 (43.4%)	6,542 (46.1%)	79,668 (44.1%)	
Missing	842 (5.3%)	9,375 (6.3%)	83 (5.5%)	735 (5.2%)	11,035 (6.1%)	
Primary location						
Left	8,912 (56.1%)	54,966 (36.8%)	524 (34.5%)	3,334 (23.5%)	67,736 (37.5%)	< 0.001
Right	6,309 (39.7%)	87,209 (58.5%)	907 (59.7%)	10,104 (71.2%)	104,529 (57.8%)	
Missing	663 (4.2%)	7,026 (4.7%)	87 (5.7%)	763 (5.4%)	8,539 (4.7%)	
Differentiation						
Poor differentiation	2,530 (15.9%)	24,086 (16.1%)	302 (19.9%)	2,689 (18.9%)	29,607 (16.4%)	< 0.001
Well differentiation	10,949 (68.9%)	103,106 (69.1%)	1,033 (68.1%)	10,050 (70.8%)	125,138 (69.2%)	
Missing	2,405 (15.1%)	22,009 (14.8%)	183 (12.1%)	1,462 (10.3%)	26,059 (14.4%)	
Stage						
I	1,624 (10.2%)	26,967 (18.1%)	92 (6.1%)	1,761 (12.4%)	30,444 (16.8%)	< 0.001
II	3,625 (22.8%)	41,204 (27.6%)	435 (28.7%)	5,132 (36.1%)	50,396 (27.9%)	
III	4,996 (31.5%)	38,936 (26.1%)	526 (34.7%)	4,289 (30.2%)	48,747 (27.0%)	
IV	5,000 (31.5%)	32,474 (21.8%)	440 (29.0%)	2,718 (19.1%)	40,632 (22.5%)	
Missing	639 (4.0%)	9,620 (6.4%)	25 (1.6%)	301 (2.1%)	10,585 (5.9%)	
T						
T1	1,276 (8.0%)	17,061 (11.4%)	40 (2.6%)	676 (4.8%)	19,053 (10.5%)	< 0.001
T2	1,270 (8.0%)	17,181 (11.5%)	102 (6.7%)	1,585 (11.2%)	20,138 (11.1%)	
T3	7,697 (48.5%)	67,960 (45.5%)	731 (48.2%)	7,376 (51.9%)	83,764 (46.3%)	
T4	3,483 (21.9%)	25,762 (17.3%)	531 (35.0%)	3,630 (25.6%)	33,406 (18.5%)	
Missing	2,158 (13.6%)	21,237 (14.2%)	114 (7.5%)	934 (6.6%)	24,443 (13.5%)	
N						
N0	6,811 (42.9%)	83,065 (55.7%)	652 (43.0%)	7,702 (54.2%)	98,230 (54.3%)	< 0.001
N1	4,544 (28.6%)	35,127 (23.5%)	451 (29.7%)	3,433 (24.2%)	43,555 (24.1%)	
N2	3,260 (20.5%)	19,306 (12.9%)	350 (23.1%)	2,523 (17.8%)	25,439 (14.1%)	
Missing	1,269 (8.0%)	11,703 (7.8%)	65 (4.3%)	543 (3.8%)	13,580 (7.5%)	
M						
M0	10,807 (68.0%)	115,993 (77.7%)	1,068 (70.4%)	11,442 (80.6%)	139,310 (77.1%)	< 0.001
M1	5,016 (31.6%)	32,568 (21.8%)	441 (29.1%)	2,718 (19.1%)	40,743 (22.5%)	
Missing	61 (0.4%)	640 (0.4%)	9 (0.6%)	41 (0.3%)	751 (0.4%)	
Surgery						
No surgery	2,419 (15.2%)	24,290 (16.3%)	134 (8.8%)	1,118 (7.9%)	27,961 (15.5%)	< 0.001
Surgery	13,391 (84.3%)	124,129 (83.2%)	1,380 (90.9%)	13,054 (91.9%)	151,954 (84.0%)	
Missing	74 (0.5%)	782 (0.5%)	4 (0.3%)	29 (0.2%)	889 (0.5%)	
Surgery/Radiation						
No radiation and/or cancer‐directed surgery	15,481 (97.5%)	147,379 (98.8%)	1,489 (98.1%)	14,017 (98.7%)	178,366 (98.7%)	< 0.001
Radiation	403 (2.5%)	1,822 (1.2%)	29 (1.9%)	184 (1.3%)	2,438 (1.3%)	
Surgery/Systemic Therapy						
Surgery	7,458 (47.0%)	108,121 (72.5%)	619 (40.8%)	9,780 (68.9%)	125,978 (69.7%)	< 0.001
Systemic Therapy	8,419 (53.0%)	41,024 (27.5%)	896 (59.0%)	4,414 (31.1%)	54,753 (30.3%)	
Missing	7 (0.0%)	56 (0.0%)	3 (0.2%)	7 (0.0%)	73 (0.0%)	
Median household income						
<$55,000	2,590 (16.3%)	27,526 (18.4%)	248 (16.3%)	2,424 (17.1%)	32,788 (18.1%)	< 0.001
<$65,000	2,763 (17.4%)	27,332 (18.3%)	290 (19.1%)	2,784 (19.6%)	33,169 (18.3%)	
<$75,000	3,769 (23.7%)	34,347 (23.0%)	405 (26.7%)	3,561 (25.1%)	42,082 (23.3%)	
>$75,000	6,762 (42.6%)	59,991 (40.2%)	575 (37.9%)	5,432 (38.3%)	72,760 (40.2%)	
Missing	0 (0%)	5 (0.0%)	0 (0%)	0 (0%)	5 (0.0%)	
Rural–urban distribution						
Metropolitan areas	9,476 (59.7%)	85,346 (57.2%)	945 (62.3%)	8,525 (60.0%)	104,292 (57.7%)	< 0.001
Intermediate areas	4,650 (29.3%)	43,017 (28.8%)	405 (26.7%)	3,844 (27.1%)	51,916 (28.7%)	
Nonmetropolitan areas	1,708 (10.8%)	20,510 (13.7%)	165 (10.9%)	1,816 (12.8%)	24,199 (13.4%)	
Missing	50 (0.3%)	328 (0.2%)	3 (0.2%)	16 (0.1%)	397 (0.2%)	

### 3.2 Pathological Characteristics

The primary location of colon cancer varied between the EOCC and LOCC groups. EOCC was located mainly on the left side of the colon (54.2%), whereas LOCC was observed primarily on the right side of the colon (59.6%). Specifically, EOAC was predominantly left‐sided (56.1%), whereas EOMC was mainly right‐sided (59.7%). Among late‐onset cases, the dominant right‐sided cohort was LOMC (71.2%). The distribution of tumor differentiation was similar between EOCC and LOCC (*p* = 0.999), although poor differentiation was notably associated with mucinous adenocarcinoma, independent of age at onset (Supplementary Table S1 and Table S2). Additionally, compared with LOCC patients, EOCC patients more commonly presented with advanced tumor stages, including more frequent T3/T4 staging, lymph node infiltration, and distant metastasis (see Table 1 for details).

### 3.3 Treatment

Overall, surgery was the main treatment for both EOCC and LOCC, but only a small proportion of patients received radiotherapy. More than half of the early‐onset patients underwent chemotherapy—53.0% of those with EOAC and 59.0% of those with EOMC. These rates were significantly greater than those of late‐onset patients: 27.5% of LOAC patients and 31.1% of LOMC patients underwent chemotherapy (see Table 1 for details).

### 3.4 Prognostic Analysis

**FIGURE 1:**
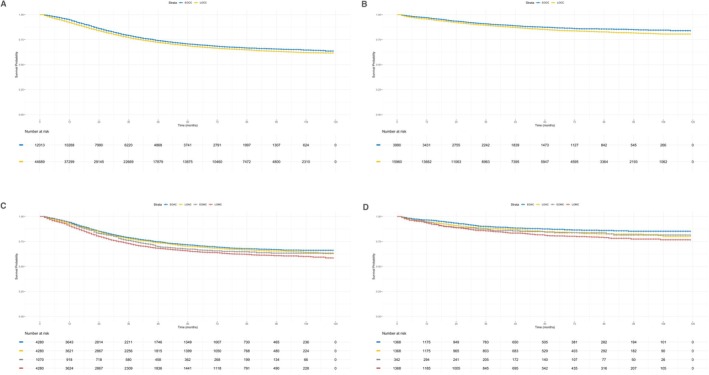
Kaplan–Meier Survival Curves for CSS and SSS in Colon Cancer Patients Stratified by Age of Onset and Histology. (A) CSS curves comparing EOCC and LOCC patients (*p* < 0.001). (B) SSS curves comparing EOCC and LOCC patients (*p* < 0.001). (C) CSS curves comparing EOAC, LOAC, EOMC, and LOMC patients, with EOAC showing the best survival and LOMC the worst (*p* < 0.001). No significant differences emerged between EOAC vs. LOAC (*p* = 0.213) or EOMC vs. LOMC (*p* = 0.077). (D) SSS curves comparing EOAC, LOAC, EOMC, and LOMC patients, with EOAC showing the best survival and LOMC the worst (*p* < 0.001). No significant differences between EOAC vs. LOAC (*p* = 0.065) or EOMC vs. LOMC (*p* = 0.288).

Initial prognostic analyses compared EOCC and LOCC without accounting for histology to assess the baseline impact of age at onset. Following PSM (Table 2), Kaplan–Meier analyses revealed significantly inferior CSS and SSS in LOCC patients than in EOCC patients (both *p* < 0.001; Figure 1A,B). PHA violations in the initial Cox proportional hazards models were addressed using piecewise and stratified modeling for CSS and SSS (global *p* = 0.056 and 0.24, respectively). These refined models confirmed a worse prognosis for LOCC: For CSS ≤ 24 months (HR 1.34, 95% CI 1.27–1.42, *p* < 0.001) and > 24 months (HR 1.16, 95% CI 1.06–1.27; *p* < 0.001), and an SSS (HR 1.35, 95% CI 1.21–1.50; *p* < 0.001) (Supplementary Table S3).

**TABLE 2:** Baseline Characteristics and Statistical Comparisons of EOCC and LOCC Patients Before and After PSM in the CSS Analysis.

Characteristics	Before PSM for CSS			After PSM for CSS			Before PSM for SSS			After PSM for SSS		
	EOCC	LOCC	*P*	EOCC	LOCC	*P*	EOCC	LOCC	*P*	EOCC	LOCC	*P*
	(*N* = 12,013)	(*N* = 63,782)		(*N* = 12,013)	(*N* = 44,689)		(*N* = 3,990)	(*N* = 33,415)		(*N* = 3,990)	(*N* = 15,960)	
Sex												
Female	5,839 (48.6%)	30,265 (47.5%)	0.067	5,839 (48.6%)	20,682 (46.3%)	< 0.001	1,900 (47.6%)	15,816 (47.3%)	0.943	1,900 (47.6%)	7,516 (47.1%)	0.837
Male	6,174 (51.4%)	33,517 (52.5%)		6,174 (51.4%)	24,007 (53.7%)		2,090 (52.4%)	17,599 (52.7%)		2,090 (52.4%)	8,444 (52.9%)	
Race												
Black	1,784 (14.9%)	8,348 (13.1%)	< 0.001	1,784 (14.9%)	6,647 (14.9%)	0.993	601 (15.1%)	4,201 (12.6%)	< 0.001	601 (15.1%)	2,339 (14.7%)	0.928
Others	1,428 (11.9%)	6,376 (10.0%)		1,428 (11.9%)	5,239 (11.7%)		494 (12.4%)	3,346 (10.0%)		494 (12.4%)	1,924 (12.1%)	
White	8,801 (73.3%)	49,058 (76.9%)		8,801 (73.3%)	32,803 (73.4%)		2,895 (72.6%)	25,868 (77.4%)		2,895 (72.6%)	11,697 (73.3%)	
Marital status												
Married	6,966 (58.0%)	38,312 (60.1%)	< 0.001	6,966 (58.0%)	25,997 (58.2%)	0.935	2,312 (57.9%)	19,928 (59.6%)	0.12	2,312 (57.9%)	9,237 (57.9%)	0.997
Umarried	5,047 (42.0%)	25,470 (39.9%)		5,047 (42.0%)	18,692 (41.8%)		1,678 (42.1%)	13,487 (40.4%)		1,678 (42.1%)	6,723 (42.1%)	
Primary location												
Left	6,699 (55.8%)	26,668 (41.8%)	< 0.001	6,699 (55.8%)	23,519 (52.6%)	< 0.001	2,063 (51.7%)	13,154 (39.4%)	< 0.001	2,063 (51.7%)	8,290 (51.9%)	0.964
Right	5,314 (44.2%)	37,114 (58.2%)		5,314 (44.2%)	21,170 (47.4%)		1,927 (48.3%)	20,261 (60.6%)		1,927 (48.3%)	7,670 (48.1%)	
Differentiation												
Well differentiation	9,710 (80.8%)	52,844 (82.9%)	< 0.001	9,710 (80.8%)	36,605 (81.9%)	0.0247	483 (12.1%)	4,029 (12.1%)	0.996	483 (12.1%)	1,821 (11.4%)	0.47
Poor differentiation	2,303 (19.2%)	10,938 (17.1%)		2,303 (19.2%)	8,084 (18.1%)		3,507 (87.9%)	29,386 (87.9%)		3,507 (87.9%)	14,139 (88.6%)	
Stage												
I	1,243 (10.3%)	12,377 (19.4%)	< 0.001	1,243 (10.3%)	4,980 (11.1%)	< 0.001	1,191 (29.8%)	12,144 (36.3%)	< 0.001	1,191 (29.8%)	4,794 (30.0%)	0.999
II	3,317 (27.6%)	19,599 (30.7%)		3,317 (27.6%)	13,520 (30.3%)		1,995 (50.0%)	15,132 (45.3%)		1,995 (50.0%)	8,019 (50.2%)	
III	4,605 (38.3%)	21,035 (33.0%)		4,605 (38.3%)	16,602 (37.2%)		604 (15.1%)	4,440 (13.3%)		604 (15.1%)	2,374 (14.9%)	
IV	2,848 (23.7%)	10,771 (16.9%)		2,848 (23.7%)	9,587 (21.5%)		200 (5.0%)	1,699 (5.1%)		200 (5.0%)	773 (4.8%)	
Median household income												
<$55,000	2,008 (16.7%)	12,436 (19.5%)	< 0.001	2,008 (16.7%)	7,749 (17.3%)	0.649	654 (16.4%)	6,214 (18.6%)	0.00679	654 (16.4%)	2,574 (16.1%)	0.986
<$65,000	2,141 (17.8%)	11,872 (18.6%)		2,141 (17.8%)	7,926 (17.7%)		719 (18.0%)	6,297 (18.8%)		719 (18.0%)	2,822 (17.7%)	
<$75,000	2,891 (24.1%)	14,420 (22.6%)		2,891 (24.1%)	10,891 (24.4%)		1,008 (25.3%)	7,772 (23.3%)		1,008 (25.3%)	4,146 (26.0%)	
>$75,000	4,973 (41.4%)	25,054 (39.3%)		4,973 (41.4%)	18,123 (40.6%)		1,609 (40.3%)	13,132 (39.3%)		1,609 (40.3%)	6,418 (40.2%)	
Rural–urban distribution												
Metropolitan areas	7,160 (59.6%)	36,270 (56.9%)	< 0.001	7,160 (59.6%)	26,572 (59.5%)	0.581	2,454 (61.5%)	19,214 (57.5%)	< 0.001	2,454 (61.5%)	9,833 (61.6%)	0.998
Intermediate areas	3,576 (29.8%)	18,625 (29.2%)		3,576 (29.8%)	13,132 (29.4%)		1,108 (27.8%)	9,620 (28.8%)		1,108 (27.8%)	4,445 (27.9%)	
Nonmetropolitan areas	1,277 (10.6%)	8,887 (13.9%)		1,277 (10.6%)	4,985 (11.2%)		428 (10.7%)	4,581 (13.7%)		428 (10.7%)	1,682 (10.5%)	

Given the differences in biological behavior and chemotherapy response between adenocarcinoma and mucinous adenocarcinoma, subsequent analyses incorporated histology to evaluate age‐at‐onset effects across the EOMC, EOAC, LOMC, and LOAC cohorts. PSM was performed between EOMC and the other groups (EOAC, LOMC, and LOAC; Tables 3 and 4) prior to survival estimation. Kaplan–Meier analyses revealed that the CSS and SSS were best for EOAC and worst for LOMC (both *p* < 0.001; Figure 1C,D). No significant differences emerged between the early‐ and late‐onset groups for adenocarcinoma (CSS: *p* = 0.213; SSS: *p* = 0.065) or mucinous adenocarcinoma (CSS: *p* = 0.077; SSS: *p* = 0.288). PHA violations in these Cox models were mitigated via piecewise and stratified approaches for CSS and SSS (global *p* = 0.074 and 0.692, respectively). According to the refined CSS model, LOMC predicted a worse prognosis than EOAC (≤ 24 months: HR 1.55, 95% CI 1.39–1.72, *p* < 0.001; > 24 months: HR 1.29, 95% CI 1.09–1.53, *p* = 0.037). Unmarried status (vs. married: HR 1.32, 95% CI 1.24–1.41; *p* < 0.001) was associated with poorer outcomes, whereas a median household income >$75,000 was protective (HR 0.83, 95% CI 0.75–0.92; *p* < 0.001) (Supplementary Table S4). In the refined SSS model, both LOMC and LOAC predicted a worse prognosis than EOAC did (HR 1.62, 95% CI 1.31–2.00, *p* < 0.001; HR 1.30, 95% CI 1.04–1.62, *p* = 0.020, respectively). Unmarried status (HR 1.39, 95% CI 1.17–1.65; *p* < 0.001) and poor differentiation (HR 1.82, 95% CI 1.51–2.20; *p* < 0.001) were linked to inferior outcomes, whereas a median household income >$75,000 remained protective (HR 0.60, 95% CI 0.46–0.77; *p* < 0.001) (Supplementary Table S4).

**TABLE 3:** Baseline Characteristics and Statistical Comparisons of Colon Cancer Patients Stratified by Age and Histology (EOAC, LOAC, EOMC, LOMC) Before and After PSM in the CSS Analysis.

Characteristics	Before PSM					After PSM					
	EOAC	LOAC	EOMC	LOMC	*P*	EOAC	LOAC	EOMC	LOMC	*P*	
	(*N* = 10,943)	(*N* = 58,369)	(*N* = 1,070)	(*N* = 5,413)		(*N* = 4,280)	(*N* = 4,280)	(*N* = 1,070)	(*N* = 4,280)		
Sex											
Female	5,420 (49.5%)	27,639 (47.4%)	419 (39.2%)	2,626 (48.5%)	< 0.001	1,708 (39.9%)	1,664 (38.9%)	419 (39.2%)	1,758 (41.1%)	0.334	
Male	5,523 (50.5%)	30,730 (52.6%)	651 (60.8%)	2,787 (51.5%)		2,572 (60.1%)	2,616 (61.1%)	651 (60.8%)	2,522 (58.9%)		
Race											
Black	1,627 (14.9%)	7,709 (13.2%)	157 (14.7%)	639 (11.8%)	< 0.001	616 (14.4%)	632 (14.8%)	157 (14.7%)	553 (12.9%)	0.047	
Others	1,314 (12.0%)	5,957 (10.2%)	114 (10.7%)	419 (7.7%)		404 (9.4%)	444 (10.4%)	114 (10.7%)	381 (8.9%)		
White	8,002 (73.1%)	44,703 (76.6%)	799 (74.7%)	4,355 (80.5%)		3,260 (76.2%)	3,204 (74.9%)	799 (74.7%)	3,346 (78.2%)		
Marital status											
Married	6,375 (58.3%)	35,104 (60.1%)	591 (55.2%)	3,208 (59.3%)	< 0.001	2,385 (55.7%)	2,383 (55.7%)	591 (55.2%)	2,469 (57.7%)	0.278	
Unmarried	4,568 (41.7%)	23,265 (39.9%)	479 (44.8%)	2,205 (40.7%)		1,895 (44.3%)	1,897 (44.3%)	479 (44.8%)	1,811 (42.3%)		
Primary location											
Left	6,321 (57.8%)	25,134 (43.1%)	378 (35.3%)	1,534 (28.3%)	< 0.001	1,487 (34.7%)	1,524 (35.6%)	378 (35.3%)	1,375 (32.1%)	0.011	
Right	4,622 (42.2%)	33,235 (56.9%)	692 (64.7%)	3,879 (71.7%)		2,793 (65.3%)	2,756 (64.4%)	692 (64.7%)	2,905 (67.9%)		
Differentiation											
Well differentiation	8,874 (81.1%)	48,504 (83.1%)	836 (78.1%)	4,340 (80.2%)	< 0.001	3,389 (79.2%)	3,374 (78.8%)	836 (78.1%)	3,398 (79.4%)	0.907	
Poor differentiation	2,069 (18.9%)	9,865 (16.9%)	234 (21.9%)	1,073 (19.8%)		891 (20.8%)	906 (21.2%)	234 (21.9%)	882 (20.6%)		
Stage											
I	1,172 (10.7%)	11,687 (20.0%)	71 (6.6%)	690 (12.7%)	< 0.001	294 (6.9%)	284 (6.6%)	71 (6.6%)	272 (6.4%)	0.582	
II	2,972 (27.2%)	17,688 (30.3%)	345 (32.2%)	1,911 (35.3%)		1,416 (33.1%)	1,384 (32.3%)	345 (32.2%)	1,486 (34.7%)		
III	4,190 (38.3%)	19,135 (32.8%)	415 (38.8%)	1,900 (35.1%)		1,604 (37.5%)	1,667 (38.9%)	415 (38.8%)	1,638 (38.3%)		
IV	2,609 (23.8%)	9,859 (16.9%)	239 (22.3%)	912 (16.8%)		966 (22.6%)	945 (22.1%)	239 (22.3%)	884 (20.7%)		
Median household income											
<$55,000	1,840 (16.8%)	11,448 (19.6%)	168 (15.7%)	988 (18.3%)	< 0.001	687 (16.1%)	655 (15.3%)	168 (15.7%)	702 (16.4%)	0.817	
<$65,000	1,933 (17.7%)	10,777 (18.5%)	208 (19.4%)	1095 (20.2%)		780 (18.2%)	815 (19.0%)	208 (19.4%)	847 (19.8%)		
<$75,000	2,613 (23.9%)	13,097 (22.4%)	278 (26.0%)	1,323 (24.4%)		1,130 (26.4%)	1,123 (26.2%)	278 (26.0%)	1,131 (26.4%)		
>$75,000	4,557 (41.6%)	23,047 (39.5%)	416 (38.9%)	2,007 (37.1%)		1,683 (39.3%)	1,687 (39.4%)	416 (38.9%)	1,600 (37.4%)		
Rural–urban distribution											
Metropolitan areas	6,494 (59.3%)	33,091 (56.7%)	666 (62.2%)	3,179 (58.7%)	< 0.001	2,652 (62.0%)	2,701 (63.1%)	666 (62.2%)	2,620 (61.2%)	0.664	
Intermediate areas	3,287 (30.0%)	17,118 (29.3%)	289 (27.0%)	1,507 (27.8%)		1,187 (27.7%)	1,149 (26.8%)	289 (27.0%)	1,174 (27.4%)		
Nonmetropolitan areas	1,162 (10.6%)	8,160 (14.0%)	115 (10.7%)	727 (13.4%)		441 (10.3%)	430 (10.0%)	115 (10.7%)	486 (11.4%)		

**TABLE 4:** Baseline Characteristics and Statistical Comparisons of Colon Cancer Patients Stratified by Age and Histology (EOAC, LOAC, EOMC, LOMC) Before and After PSM in the SSS Analysis.

Characteristics	Before PSM					After PSM					
	EOAC	LOAC	EOMC	LOMC	*P*	EOAC	LOAC	EOMC	LOMC	*P*	
	(*N* = 3,648)	(*N* = 30,681)	(*N* = 342)	(*N* = 2,734)		(*N* = 1,368)	(*N* = 1,368)	(*N* = 342)	(*N* = 1,368)		
Sex											
Female	1,770 (48.5%)	14,494 (47.2%)	130 (38.0%)	1,322 (48.4%)	0.00428	525 (38.4%)	514 (37.6%)	130 (38.0%)	527 (38.5%)	0.989	
Male	1,878 (51.5%)	16,187 (52.8%)	212 (62.0%)	1,412 (51.6%)		843 (61.6%)	854 (62.4%)	212 (62.0%)	841 (61.5%)		
Race											
Black	545 (14.9%)	3,891 (12.7%)	56 (16.4%)	310 (11.3%)	< 0.001	199 (14.5%)	221 (16.2%)	56 (16.4%)	219 (16.0%)	0.946	
Others	460 (12.6%)	3,129 (10.2%)	34 (9.9%)	217 (7.9%)		122 (8.9%)	125 (9.1%)	34 (9.9%)	133 (9.7%)		
White	2,643 (72.5%)	23,661 (77.1%)	252 (73.7%)	2,207 (80.7%)		1,047 (76.5%)	1,022 (74.7%)	252 (73.7%)	1,016 (74.3%)		
Marital status											
Married	2,138 (58.6%)	18,361 (59.8%)	174 (50.9%)	1,567 (57.3%)	< 0.001	677 (49.5%)	715 (52.3%)	174 (50.9%)	720 (52.6%)	0.512	
Unmarried	1,510 (41.4%)	12,320 (40.2%)	168 (49.1%)	1,167 (42.7%)		691 (50.5%)	653 (47.7%)	168 (49.1%)	648 (47.4%)		
Primary location											
Left	1,963 (53.8%)	12,414 (40.5%)	100 (29.2%)	740 (27.1%)	< 0.001	388 (28.4%)	384 (28.1%)	100 (29.2%)	395 (28.9%)	0.988	
Right	1,685 (46.2%)	18,267 (59.5%)	242 (70.8%)	1,994 (72.9%)		980 (71.6%)	984 (71.9%)	242 (70.8%)	973 (71.1%)		
Differentiation											
Well differentiation	3,214 (88.1%)	27,044 (88.1%)	293 (85.7%)	2,342 (85.7%)	0.00259	1,202 (87.9%)	1,197 (87.5%)	293 (85.7%)	1,188 (86.8%)	0.824	
Poor differentiation	434 (11.9%)	3,637 (11.9%)	49 (14.3%)	392 (14.3%)		166 (12.1%)	171 (12.5%)	49 (14.3%)	180 (13.2%)		
Stage											
I	1,123 (30.8%)	11,464 (37.4%)	68 (19.9%)	680 (24.9%)	< 0.001	288 (21.1%)	278 (20.3%)	68 (19.9%)	267 (19.5%)	0.873	
II	1,797 (49.3%)	13,663 (44.5%)	198 (57.9%)	1,469 (53.7%)		820 (59.9%)	793 (58.0%)	198 (57.9%)	817 (59.7%)		
III	552 (15.1%)	4,040 (13.2%)	52 (15.2%)	400 (14.6%)		192 (14.0%)	208 (15.2%)	52 (15.2%)	193 (14.1%)		
IV	176 (4.8%)	1,514 (4.9%)	24 (7.0%)	185 (6.8%)		68 (5.0%)	89 (6.5%)	24 (7.0%)	91 (6.7%)		
Median household income											
<$55,000	598 (16.4%)	5,739 (18.7%)	56 (16.4%)	475 (17.4%)	< 0.001	241 (17.6%)	229 (16.7%)	56 (16.4%)	222 (16.2%)	0.99	
<$65,000	659 (18.1%)	5,741 (18.7%)	60 (17.5%)	556 (20.3%)		252 (18.4%)	237 (17.3%)	60 (17.5%)	228 (16.7%)		
<$75,000	912 (25.0%)	7,067 (23.0%)	96 (28.1%)	705 (25.8%)		369 (27.0%)	392 (28.7%)	96 (28.1%)	387 (28.3%)		
>$75,000	1,479 (40.5%)	12,134 (39.5%)	130 (38.0%)	998 (36.5%)		506 (37.0%)	510 (37.3%)	130 (38.0%)	531 (38.8%)		
Rural–urban distribution											
Metropolitan areas	2,234 (61.2%)	17,562 (57.2%)	220 (64.3%)	1,652 (60.4%)	< 0.001	840 (61.4%)	893 (65.3%)	220 (64.3%)	902 (65.9%)	0.503	
Intermediate areas	1,028 (28.2%)	8,879 (28.9%)	80 (23.4%)	741 (27.1%)		355 (26.0%)	317 (23.2%)	80 (23.4%)	314 (23.0%)		
Nonmetropolitan areas	386 (10.6%)	4,240 (13.8%)	42 (12.3%)	341 (12.5%)		173 (12.6%)	158 (11.5%)	42 (12.3%)	152 (11.1%)		

### 3.5 Prognostic Analysis Stratified by Distant Metastasis

Colon cancer management differs by metastatic status: Patients with stage IV disease primarily receive systemic or targeted therapies, whereas those with stages I–III disease undergo surgery. Baseline characteristics revealed significant differences in distant metastasis distribution between the early‐onset (EOAC and EOMC) and late‐onset (LOAC and LOMC) groups (Table 1). Cox models revealed a significant interaction between histology‐age group and stage (concordance index 0.787, *p* < 0.001 for CSS; concordance index 0.781, *p* = 0.022 for SSS), prompting subgroup analyses stratified by distant metastasis. PSM was performed between EOMC and the other cohorts (EOAC, LOAC, and LOMC) prior to survival estimation (Tables 5, 6, 7, 8).

**TABLE 5:** Baseline Characteristics and Statistical Comparisons of Nonmetastatic Colon Cancer Patients Before and After PSM in the CSS Analysis.

Characteristics	Before PSM					After PSM				
	EOAC	LOAC	EOMC	LOMC	*P*	EOAC	LOAC	EOMC	LOMC	*P*
	(*N* = 8,334)	(*N* = 48,510)	(*N* = 831)	(*N* = 4,501)		(*N* = 3,324)	(*N* = 3,324)	(*N* = 831)	(*N* = 3,324)	
Sex										
Female	4,096 (49.1%)	23,191 (47.8%)	310 (37.3%)	2,172 (48.3%)	< 0.001	1,210 (36.4%)	1,240 (37.3%)	310 (37.3%)	1,231 (37.0%)	0.956
Male	4,238 (50.9%)	25,319 (52.2%)	521 (62.7%)	2,329 (51.7%)		2,114 (63.6%)	2,084 (62.7%)	521 (62.7%)	2,093 (63.0%)	
Race										
Black	1,229 (14.7%)	6,193 (12.8%)	112 (13.5%)	509 (11.3%)	< 0.001	467 (14.0%)	463 (13.9%)	112 (13.5%)	420 (12.6%)	0.335
Others	1,010 (12.1%)	5,046 (10.4%)	90 (10.8%)	356 (7.9%)		333 (10.0%)	345 (10.4%)	90 (10.8%)	300 (9.0%)	
White	6,095 (73.1%)	37,271 (76.8%)	629 (75.7%)	3,636 (80.8%)		2,524 (75.9%)	2,516 (75.7%)	629 (75.7%)	2,604 (78.3%)	
Marital status										
Married	4,876 (58.5%)	29,472 (60.8%)	468 (56.3%)	2,707 (60.1%)	< 0.001	1,894 (57.0%)	1,891 (56.9%)	468 (56.3%)	1,959 (58.9%)	0.386
Unmarried	3,458 (41.5%)	19,038 (39.2%)	363 (43.7%)	1,794 (39.9%)		1,430 (43.0%)	1,433 (43.1%)	363 (43.7%)	1,365 (41.1%)	
Primary location										
Left	4,742 (56.9%)	20,675 (42.6%)	288 (34.7%)	1,235 (27.4%)	< 0.001	1,151 (34.6%)	1,138 (34.2%)	288 (34.7%)	1,162 (35.0%)	0.984
Right	3,592 (43.1%)	27,835 (57.4%)	543 (65.3%)	3,266 (72.6%)		2,173 (65.4%)	2,186 (65.8%)	543 (65.3%)	2,162 (65.0%)	
Differentiation										
Well differentiation	6,924 (83.1%)	41,230 (85.0%)	684 (82.3%)	3,695 (82.1%)	< 0.001	2,760 (83.0%)	2,758 (83.0%)	684 (82.3%)	2,774 (83.5%)	0.95
Poor differentiation	1,410 (16.9%)	7,280 (15.0%)	147 (17.7%)	806 (17.9%)		564 (17.0%)	566 (17.0%)	147 (17.7%)	550 (16.5%)	
Median household income										
<$55,000	1,397 (16.8%)	9,323 (19.2%)	128 (15.4%)	814 (18.1%)	< 0.001	525 (15.8%)	502 (15.1%)	128 (15.4%)	508 (15.3%)	0.702
<$65,000	1,443 (17.3%)	8,865 (18.3%)	159 (19.1%)	922 (20.5%)		593 (17.8%)	653 (19.6%)	159 (19.1%)	676 (20.3%)	
<$75,000	1,966 (23.6%)	10,862 (22.4%)	220 (26.5%)	1,063 (23.6%)		858 (25.8%)	882 (26.5%)	220 (26.5%)	864 (26.0%)	
>$75,000	3,528 (42.3%)	19,460 (40.1%)	324 (39.0%)	1,702 (37.8%)		1,348 (40.6%)	1,287 (38.7%)	324 (39.0%)	1,276 (38.4%)	
Rural–urban distribution										
Metropolitan areas	4,966 (59.6%)	27,603 (56.9%)	515 (62.0%)	2,658 (59.1%)	< 0.001	2,065 (62.1%)	2,061 (62.0%)	515 (62.0%)	2,037 (61.3%)	0.987
Intermediate areas	2,465 (29.6%)	14,169 (29.2%)	222 (26.7%)	1,245 (27.7%)		889 (26.7%)	908 (27.3%)	222 (26.7%)	901 (27.1%)	
Nonmetropolitan areas	903 (10.8%)	6,738 (13.9%)	94 (11.3%)	598 (13.3%)		370 (11.1%)	355 (10.7%)	94 (11.3%)	386 (11.6%)	

**TABLE 6:** Baseline Characteristics and Statistical Comparisons of Nonmetastatic Colon Cancer Patients Before and After PSM in the SSS Analysis.

Characteristics	Before PSM					After PSM				
	EOAC	LOAC	EOMC	LOMC	*P*	EOAC	LOAC	EOMC	LOMC	*P*
	(*N* = 3,472)	(*N* = 29,167)	(*N* = 318)	(*N* = 2,549)		(*N* = 1,272)	(*N* = 1,272)	(*N* = 318)	(*N* = 1,272)	
Sex										
Female	1,684 (48.5%)	13,835 (47.4%)	120 (37.7%)	1,219 (47.8%)	0.00829	477 (37.5%)	482 (37.9%)	120 (37.7%)	481 (37.8%)	1
Male	1,788 (51.5%)	15,332 (52.6%)	198 (62.3%)	1,330 (52.2%)		795 (62.5%)	790 (62.1%)	198 (62.3%)	791 (62.2%)	
Race										
Black	509 (14.7%)	3,643 (12.5%)	48 (15.1%)	281 (11.0%)	< 0.001	176 (13.8%)	189 (14.9%)	48 (15.1%)	200 (15.7%)	0.613
Others	441 (12.7%)	2,974 (10.2%)	32 (10.1%)	203 (8.0%)		96 (7.5%)	121 (9.5%)	32 (10.1%)	107 (8.4%)	
White	2,522 (72.6%)	22,550 (77.3%)	238 (74.8%)	2,065 (81.0%)		1,000 (78.6%)	962 (75.6%)	238 (74.8%)	965 (75.9%)	
Marital status										
Married	2,068 (59.6%)	17,644 (60.5%)	167 (52.5%)	1,500 (58.8%)	0.0222	680 (53.5%)	670 (52.7%)	167 (52.5%)	674 (53.0%)	0.996
Unmarried	1,404 (40.4%)	11,523 (39.5%)	151 (47.5%)	1,049 (41.2%)		592 (46.5%)	602 (47.3%)	151 (47.5%)	598 (47.0%)	
Primary location										
Left	1,861 (53.6%)	11,788 (40.4%)	95 (29.9%)	681 (26.7%)	< 0.001	379 (29.8%)	374 (29.4%)	95 (29.9%)	403 (31.7%)	0.77
Right	1,611 (46.4%)	17,379 (59.6%)	223 (70.1%)	1,868 (73.3%)		893 (70.2%)	898 (70.6%)	223 (70.1%)	869 (68.3%)	
Differentiation										
Well differentiation	3,085 (88.9%)	25,960 (89.0%)	273 (85.8%)	2,214 (86.9%)	0.00832	1,103 (86.7%)	1,096 (86.2%)	273 (85.8%)	1,117 (87.8%)	0.763
Poor differentiation	387 (11.1%)	3,207 (11.0%)	45 (14.2%)	335 (13.1%)		169 (13.3%)	176 (13.8%)	45 (14.2%)	155 (12.2%)	
Median household income										
<$55,000	568 (16.4%)	5,413 (18.6%)	52 (16.4%)	440 (17.3%)	< 0.001	204 (16.0%)	204 (16.0%)	52 (16.4%)	202 (15.9%)	1
<$65,000	616 (17.7%)	5,408 (18.5%)	56 (17.6%)	526 (20.6%)		221 (17.4%)	224 (17.6%)	56 (17.6%)	223 (17.5%)	
<$75,000	864 (24.9%)	6,684 (22.9%)	86 (27.0%)	637 (25.0%)		331 (26.0%)	342 (26.9%)	86 (27.0%)	346 (27.2%)	
>$75,000	1,424 (41.0%)	11,662 (40.0%)	124 (39.0%)	946 (37.1%)		516 (40.6%)	502 (39.5%)	124 (39.0%)	501 (39.4%)	
Rural–urban distribution										
Metropolitan areas	2,135 (61.5%)	16,728 (57.4%)	202 (63.5%)	1,538 (60.3%)	< 0.001	785 (61.7%)	812 (63.8%)	202 (63.5%)	807 (63.4%)	0.951
Intermediate areas	969 (27.9%)	8,421 (28.9%)	76 (23.9%)	686 (26.9%)		330 (25.9%)	300 (23.6%)	76 (23.9%)	318 (25.0%)	
Nonmetropolitan areas	368 (10.6%)	4,018 (13.8%)	40 (12.6%)	325 (12.8%)		157 (12.3%)	160 (12.6%)	40 (12.6%)	147 (11.6%)	

**TABLE 7:** Baseline Characteristics and Statistical Comparisons of Metastatic Colon Cancer Patients Before and After PSM in the CSS Analysis.

Characteristics	Before PSM					After PSM				
	EOAC	LOAC	EOMC	LOMC	*P*	EOAC	LOAC	EOMC	LOMC	*P*
	(*N* = 2,609)	(*N* = 9,859)	(*N* = 239)	(*N* = 912)		(*N* = 956)	(*N* = 956)	(*N* = 239)	(*N* = 912)	
Sex										
Female	1,324 (50.7%)	4,448 (45.1%)	109 (45.6%)	454 (49.8%)	< 0.001	422 (44.1%)	424 (44.4%)	109 (45.6%)	454 (49.8%)	0.106
Male	1,285 (49.3%)	5,411 (54.9%)	130 (54.4%)	458 (50.2%)		534 (55.9%)	532 (55.6%)	130 (54.4%)	458 (50.2%)	
Race										
Black	398 (15.3%)	1,516 (15.4%)	45 (18.8%)	130 (14.3%)	0.00102	179 (18.7%)	181 (18.9%)	45 (18.8%)	130 (14.3%)	0.048
Others	304 (11.7%)	911 (9.2%)	24 (10.0%)	63 (6.9%)		66 (6.9%)	84 (8.8%)	24 (10.0%)	63 (6.9%)	
White	1,907 (73.1%)	7,432 (75.4%)	170 (71.1%)	719 (78.8%)		711 (74.4%)	691 (72.3%)	170 (71.1%)	719 (78.8%)	
Marital status										
Married	1,499 (57.5%)	5,632 (57.1%)	123 (51.5%)	501 (54.9%)	0.304	509 (53.2%)	504 (52.7%)	123 (51.5%)	501 (54.9%)	0.843
Unmarried	1,110 (42.5%)	4,227 (42.9%)	116 (48.5%)	411 (45.1%)		447 (46.8%)	452 (47.3%)	116 (48.5%)	411 (45.1%)	
Primary location										
Left	1,579 (60.5%)	4,459 (45.2%)	90 (37.7%)	299 (32.8%)	< 0.001	350 (36.6%)	354 (37.0%)	90 (37.7%)	299 (32.8%)	0.302
Right	1,030 (39.5%)	5,400 (54.8%)	149 (62.3%)	613 (67.2%)		606 (63.4%)	602 (63.0%)	149 (62.3%)	613 (67.2%)	
Differentiation										
Well differentiation	1,950 (74.7%)	7,274 (73.8%)	152 (63.6%)	645 (70.7%)	0.00118	625 (65.4%)	609 (63.7%)	152 (63.6%)	645 (70.7%)	0.017
Poor differentiation	659 (25.3%)	2,585 (26.2%)	87 (36.4%)	267 (29.3%)		331 (34.6%)	347 (36.3%)	87 (36.4%)	267 (29.3%)	
Median household income										
<$55,000	443 (17.0%)	2,125 (21.6%)	40 (16.7%)	174 (19.1%)	< 0.001	164 (17.2%)	153 (16.0%)	40 (16.7%)	174 (19.1%)	0.493
<$65,000	490 (18.8%)	1,912 (19.4%)	49 (20.5%)	173 (19.0%)		187 (19.6%)	198 (20.7%)	49 (20.5%)	173 (19.0%)	
<$75,000	647 (24.8%)	2,235 (22.7%)	58 (24.3%)	260 (28.5%)		234 (24.5%)	241 (25.2%)	58 (24.3%)	260 (28.5%)	
>$75,000	1,029 (39.4%)	3,587 (36.4%)	92 (38.5%)	305 (33.4%)		371 (38.8%)	364 (38.1%)	92 (38.5%)	305 (33.4%)	
Rural–urban distribution										
Metropolitan areas	1,528 (58.6%)	5,488 (55.7%)	151 (63.2%)	521 (57.1%)	< 0.001	596 (62.3%)	605 (63.3%)	151 (63.2%)	521 (57.1%)	0.006
Intermediate areas	822 (31.5%)	2,949 (29.9%)	67 (28.0%)	262 (28.7%)		272 (28.5%)	271 (28.3%)	67 (28.0%)	262 (28.7%)	
Nonmetropolitan areas	259 (9.9%)	1,422 (14.4%)	21 (8.8%)	129 (14.1%)		88 (9.2%)	80 (8.4%)	21 (8.8%)	129 (14.1%)	

**TABLE 8:** Baseline Characteristics and Statistical Comparisons of Metastatic Colon Cancer Patients Before and After PSM in the SSS Analysis.

Characteristics	Before PSM					After PSM				
	EOAC	LOAC	EOMC	LOMC	*P*	EOAC	LOAC	EOMC	LOMC	*P*
	(*N* = 176)	(*N* = 1,514)	(*N* = 24)	(*N* = 185)		(*N* = 96)	(*N* = 96)	(*N* = 24)	(*N* = 96)	
Sex										
Female	86 (48.9%)	659 (43.5%)	10 (41.7%)	103 (55.7%)	0.0268	50 (52.1%)	42 (43.8%)	10 (41.7%)	46 (47.9%)	0.794
Male	90 (51.1%)	855 (56.5%)	14 (58.3%)	82 (44.3%)		46 (47.9%)	54 (56.3%)	14 (58.3%)	50 (52.1%)	
Race										
Black	36 (20.5%)	248 (16.4%)	8 (33.3%)	29 (15.7%)	0.392	29 (30.2%)	32 (33.3%)	8 (33.3%)	25 (26.0%)	0.986
Others	19 (10.8%)	155 (10.2%)	2 (8.3%)	14 (7.6%)		9 (9.4%)	7 (7.3%)	2 (8.3%)	7 (7.3%)	
White	121 (68.8%)	1,111 (73.4%)	14 (58.3%)	142 (76.8%)		58 (60.4%)	57 (59.4%)	14 (58.3%)	64 (66.7%)	
Marital status										
Married	70 (39.8%)	717 (47.4%)	7 (29.2%)	67 (36.2%)	0.00933	27 (28.1%)	39 (40.6%)	7 (29.2%)	26 (27.1%)	0.276
Unmarried	106 (60.2%)	797 (52.6%)	17 (70.8%)	118 (63.8%)		69 (71.9%)	57 (59.4%)	17 (70.8%)	70 (72.9%)	
Primary location										
Left	102 (58.0%)	626 (41.3%)	5 (20.8%)	59 (31.9%)	< 0.001	32 (33.3%)	14 (14.6%)	5 (20.8%)	22 (22.9%)	0.049
Right	74 (42.0%)	888 (58.7%)	19 (79.2%)	126 (68.1%)		64 (66.7%)	82 (85.4%)	19 (79.2%)	74 (77.1%)	
Differentiation										
Well differentiation	129 (73.3%)	1,084 (71.6%)	20 (83.3%)	128 (69.2%)	0.662	74 (77.1%)	77 (80.2%)	20 (83.3%)	73 (76.0%)	0.920
Poor differentiation	47 (26.7%)	430 (28.4%)	4 (16.7%)	57 (30.8%)		22 (22.9%)	19 (19.8%)	4 (16.7%)	23 (24.0%)	
Median household income										
<$55,000	30 (17.0%)	326 (21.5%)	4 (16.7%)	35 (18.9%)	0.169	18 (18.8%)	14 (14.6%)	4 (16.7%)	18 (18.8%)	0.982
<$65,000	43 (24.4%)	333 (22.0%)	4 (16.7%)	30 (16.2%)		18 (18.8%)	18 (18.8%)	4 (16.7%)	12 (12.5%)	
<$75,000	48 (27.3%)	383 (25.3%)	10 (41.7%)	68 (36.8%)		33 (34.4%)	42 (43.8%)	10 (41.7%)	38 (39.6%)	
>$75,000	55 (31.3%)	472 (31.2%)	6 (25.0%)	52 (28.1%)		27 (28.1%)	22 (22.9%)	6 (25.0%)	28 (29.2%)	
Rural–urban distribution										
Metropolitan areas	99 (56.3%)	834 (55.1%)	18 (75.0%)	114 (61.6%)	0.177	64 (66.7%)	74 (77.1%)	18 (75.0%)	72 (75.0%)	0.655
Intermediate areas	59 (33.5%)	458 (30.3%)	4 (16.7%)	55 (29.7%)		22 (22.9%)	16 (16.7%)	4 (16.7%)	12 (12.5%)	
Nonmetropolitan areas	18 (10.2%)	222 (14.7%)	2 (8.3%)	16 (8.6%)		10 (10.4%)	6 (6.3%)	2 (8.3%)	12 (12.5%)	

**FIGURE 2:**
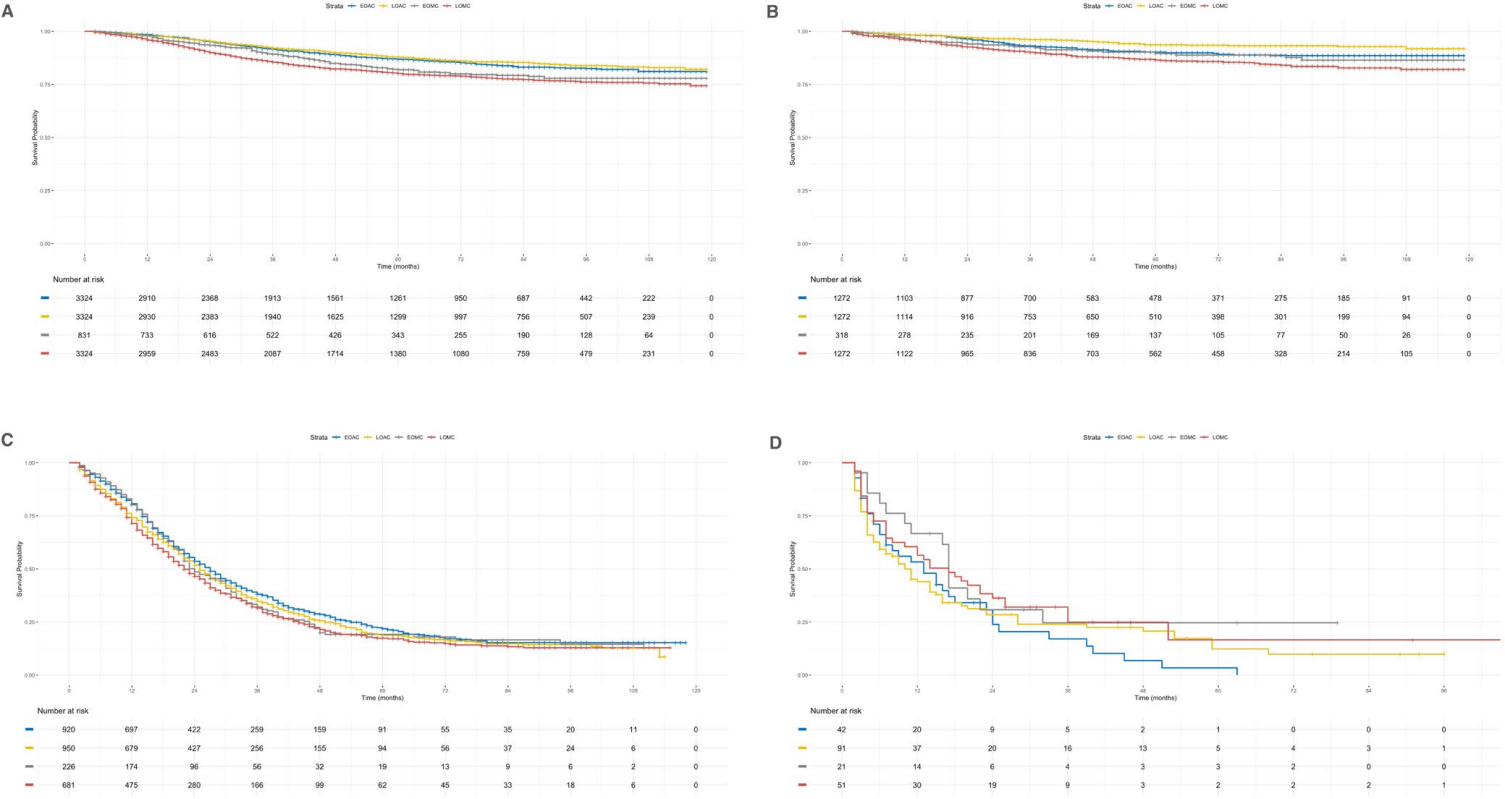
Kaplan–Meier Survival Curves for CSS and SSS in Colon Cancer Patients Stratified by Metastatic Status. (A) CSS in the nonmetastatic subgroup, with the best survival for LOAC and the worst for LOMC (*p* < 0.001). No significant differences between EOAC vs. LOAC (*p* = 0.284) or EOMC vs. LOMC (*p* = 0.127). (B) SSS in the nonmetastatic subgroup, with the best survival for LOAC and the worst for LOMC (*p* < 0.001). No significant differences between EOMC vs. LOMC (*p* = 0.204). LOAC had superior SSS compared to EOAC (*p* = 0.008). (C) CSS in the metastatic subgroup, with the best survival for EOAC and the worst for LOMC (*p* = 0.003). No significant differences between EOAC vs. LOAC (*p* = 0.210) or EOMC vs. LOMC (*p* = 0.347). (D) SSS in the metastatic subgroup, showing no significant differences among groups (*p* = 0.39).

In the nonmetastatic subgroup (stages I–III), Kaplan–Meier analyses indicated that CSS and SSS were best for LOAC and worst for LOMC (both *p* < 0.001; Figure 2A,B). No significant differences were observed between EOMC and LOMC (CSS: *p* = 0.127; SSS: *p* = 0.204). Notably, LOAC and EOAC had a comparable CSS (*p* = 0.284), but LOAC had a superior SSS (*p* = 0.008). The CSS Cox model was refined by stratifying by differentiation to ensure PHA adherence (global *p* = 0.095). Compared with EOAC, LOMC predicted a worse prognosis (≤ 24 months: HR 2.14, 95% CI 1.75–2.61, *p* < 0.001; > 24 months: HR 1.23, 95% CI 1.11–1.62, *p* < 0.001), whereas LOAC and EOAC did not differ in prognosis (≤ 24 months: HR 0.96, 95% CI 0.76–1.21, *p* = 0.719; > 24 months: HR 0.88, 95% CI 0.65–1.19, *p* = 0.591). Additional predictors of worse CSS included male sex (HR 1.14, 95% CI 1.02–1.27; *p* = 0.026) and being unmarried (HR 1.35, 95% CI 1.21–1.50; *p* < 0.001); protective factors included being white (vs. Black: HR 0.85, 95% CI 0.73–0.99; *p* = 0.034) and having a median household income > $75,000 (vs. < $55,000: HR 0.76, 95% CI 0.65–0.89; *p* < 0.001). The SSS Cox model in which PHA was satisfied (global *p* = 0.52) confirmed that EOMC had the worst prognosis (HR 1.57, 95% CI 1.21–2.03; *p* < 0.001) and that LOAC had a better prognosis than EOAC did (HR 0.61, 95% CI 0.44–0.84; *p* = 0.003). Unmarried status (HR 1.38; 95% CI 1.11–1.72; *p* = 0.004) and poor differentiation (HR 2.74; 95% CI 2.15–3.49; *p* < 0.001) were associated with inferior outcomes, whereas a median household income > $75,000 was protective (vs. < $55,000; HR 0.57; 95% CI 0.41–0.79; *p* < 0.001) (see Supplementary Table S5 for details).

In the metastatic subgroup (stage IV), Kaplan–Meier analyses revealed that CSS was best among patients with EOAC and worst among those with LOMC (*p* = 0.003; Figure 2C). No significant differences emerged between the early‐ and late‐onset groups for adenocarcinoma (*p* = 0.210) or mucinous adenocarcinoma (*p* = 0.347). SSS did not significantly differ among the groups (*p* = 0.39; Figure 2D). The refined CSS Cox model, stratified by primary location and differentiation for PHA adherence (global *p* = 0.052), revealed LOMC (vs. EOAC: HR 1.23, 95% CI 1.09–1.39, *p* < 0.001) and unmarried status (vs. married: HR 1.30, 95% CI 1.19–1.43, *p* < 0.001) as predictors of worse outcomes. The SSS Cox model, which satisfied the PHA (global *p* = 0.59) and included only marital status and differentiation, identified poor differentiation as a predictor of worse SSS (HR 2.25, 95% CI 1.57–3.23; *p* < 0.001) (see Supplementary Table S6 for details).

## 4 Discussion

The incidence of EOCRC is increasing worldwide, drawing significant attention from clinicians and researchers who have investigated its clinicopathological characteristics, molecular features, and prognosis. Consensus has emerged on distinctive EOCRC traits, such as advanced stage, aggressive histopathology, and distal location [5]. In this study, colon cancer was analyzed independently rather than in combination with rectal cancer, which minimized the confounding effects of anatomical and therapeutic differences. Both EOCC and LOCC exhibited male predominance; for EOCC, this predominance was driven primarily by the EOMC subgroup (59.4%). In contrast, the LOMC group showed female predominance. EOCC also displayed racial disparities, occurring relatively more frequently in black patients and those of other races than in white patients (*p* < 0.001). These findings align with prior reports on EOCRC incidence patterns by sex and race [10, 11].

Previous studies have demonstrated that younger patients have a greater incidence of colorectal cancer in the rectum and distal colon than in the proximal colon [11, 12, 13]. Consistent with this fact, EOAC was predominantly left‐sided (56.1%) in this study, whereas EOMC was mainly right‐sided (59.7%). The relationship between primary tumor location and age at onset appears to be histology dependent. As reported in other EOCRC studies, EOCC was associated with more advanced stages than LOCC, including greater rates of stage T3/T4 disease, lymph node involvement, and distant metastasis. However, the tumor differentiation distributions did not significantly differ among the four groups (EOAC, EOMC, LOAC, and LOMC; *p* = 0.999).

Compared with LOCC, EOCC has distinct differences in demographic distribution and clinicopathological features. Prognosis remains a primary focus in comparisons between EOCC and LOCC. Although EOCRC is predicted to have a poor prognosis because of its unfavorable histopathological features, most studies report survival outcomes comparable to those of LOCRC [5, 6]. One potential explanation is that younger patients may possess greater physical resilience and tolerance to treatment‐related toxicity [4]. In this study, survival analyses indicated that compared with LOCC, EOCC had a better prognosis overall. Specifically, CSS and SSS were best for EOAC and worst for LOMC. However, the oncological outcomes were comparable between EOCC and LOCC when patients were stratified by histology (adenocarcinoma vs. mucinous adenocarcinoma). These results suggest that differences in prognosis between EOCC and LOCC are influenced by tumor biology, particularly the differences between adenocarcinoma and mucinous subtypes. Subset analyses in patients with nonmetastatic colon cancer revealed no significant differences in CSS or SSS between EOMC and LOMC. In contrast, although the CSS was comparable between EOAC and LOAC, SSS was better for LOAC than for EOAC. These results reaffirm the well‐established influence of histological type on prognosis, as mucinous histology is often associated with distinct biological behaviors and treatment responses.

The difference between the SSS and CSS in nonmetastatic colon adenocarcinoma is particularly noteworthy. SSS, which adjusts for the confounding effects of adjuvant therapies, suggests that EOAC may have inherently poorer prognoses, which is likely attributable to aggressive tumor biology, such as rapid local progression or elevated recurrence rates. In contrast, CSS, which incorporates systemic therapy effects, was not significantly different. This disparity could be attributed to better drug tolerability in younger patients, who often possess superior physiological reserve and fewer comorbidities, enabling more aggressive treatments that mitigate survival differences. In metastatic patients, who primarily receive comprehensive systemic therapy, CSS was prioritized as the primary endpoint. Here, CSS was better for EOAC than LOAC but did not differ between EOMC and LOMC. This pattern further supports the hypothesis that improved drug tolerability in younger patients improves outcomes, particularly in adenocarcinoma subtypes, for which targeted therapies and chemotherapy play a pivotal role [5, 14]. These results emphasize that prognostic differences between EOCC and LOCC are not solely attributable to intrinsic aggressive behaviors in EOCC but are heavily influenced by confounding factors such as stage at diagnosis and histological subtype. Instead, stratified and subset analyses suggest that age at onset exerts a limited and subtle influence on prognosis, which is readily modulated by factors such as histological type, disease stage, and systemic therapy.

The prognosis of EOCRC remains complex and often conflicting compared with that of LOCRC. For instance, a population‐based study of 34,434 patients with colorectal cancer using DFS as the endpoint revealed that for stages I–III, stage‐adjusted DFS was better among younger patients than among older patients [15]. Additionally, younger patients were also more likely to receive adjuvant chemotherapy [15]. Similarly, a nationwide survey across 19 hospitals involving 991 EOCRC patients and 3581 LOCRC patients revealed that despite more aggressive features and intensified treatment in EOCRC patients, health‐related quality of life was similar between groups [16]. In contrast, a retrospective analysis from six international tertiary cancer centers, which included 498 patients with colorectal adenocarcinoma, indicated that progression‐free survival was longer among LOCRC patients (aged > 44 years) [17]. Another study of 4468 patients demonstrated worse 3‐year DFS among EOCRC patients than among LOCRC patients, despite better 3‐year overall survival among EOCRC patients [8]. A systemic review of 37 articles on EOCRC prognosis further highlighted age‐specific variations, with worse survival in patients younger than 30 years but comparable or better outcomes in those aged 40–50 years than in those older than 50 years [14]. Overall, no consensus exists on whether EOCRC confers a more favorable or worse oncological outcome than LOCRC does because the results across studies remain highly inconsistent.

In addition to the pathological and prognostic differences between EOCC and LOCC, this study highlights that marital status and median household income significantly influence oncological outcomes in patients with colon cancer. Unmarried patients had worse prognoses than married patients did, which aligns with systematic reviews that underscore the importance of social support in cancer care [18]. Furthermore, patients with higher median household incomes had better outcomes than those with lower incomes did, potentially because of disparities in care quality [19]. A positive dose–response relationship between household income and health‐related quality of life (HRQoL) has been observed in patients with various malignancies, including colon cancer [20]. Notably, a significant positive correlation exists between the time elapsed from diagnosis or relapse and HRQoL among young adults with colorectal cancer [21].

To improve the understanding of EOCRC, genetic and immunological features have been explored. For example, defective mismatch repair is more prevalent in EOCRC, as are mutations in PIK3CA and KRAS, particularly in left‐sided colon cancers [12]. Studies have also shown that compared with older patients (≥ 50 years), younger patients (< 40 years) with colorectal cancer harbor more TP53 and CTNNB1 mutations but fewer APC, KRAS, BRAF, and FAM123B mutations [22]. These genetic alterations are relevant to tumor biology and therapeutic response in colorectal cancer, although findings concerning the molecular features of EOCRC remain inconclusive. In contrast, immunological profiles consistently indicated lower levels of tumor‐infiltrating lymphocytes and peritumoral lymphocytic reactions in EOCRC than in LOCRC [23, 24].

This study has several limitations that warrant consideration. First, prognostic factors known to potentially influence survival in patients with colon cancer—such as body mass index (BMI), performance status, and hereditary cancer syndromes—were unavailable in the SEER database, potentially introducing residual confounding. Second, the limited sample size of patient cohorts derived from PSM based on EOMC may limit statistical power and introduce selection bias in subset analyses. Third, the absence of recurrence data precluded the assessment of progression‐ or recurrence‐free survival, which are more sensitive indicators of tumor biology and treatment response than overall survival metrics alone. Despite these limitations, this study offers notable strengths that enhance its contributions to the field. By focusing exclusively on colon cancer and excluding rectal cancer, the analysis minimized anatomical and therapeutic confounding (e.g., differences in neoadjuvant approaches), enabling a more precise comparison between EOCC and LOCC. Furthermore, analyses stratified by histology and tumor stage provided refined insights into age‐related prognostic differences, revealing nuances often obscured in unadjusted studies. Finally, the dual use of CSS and SSS as endpoints allowed for a comprehensive evaluation of prognoses under varying treatment contexts, offering a clearer understanding of intrinsic vs. modifiable factors in nonmetastatic colon cancer.

In conclusion, this study demonstrated that the demographic distribution and clinicopathological features of EOCC differ from those of LOCC. Prognostic analyses comparing EOCC and LOCC are complex, and accounting for the influence of tumor stage and histology is essential. To more precisely evaluate the impact of age at onset on the malignant behavior of patients with colon cancer, future studies should incorporate additional covariates (e.g., BMI and HRQoL) and progression/relapse‐related prognostic indicators. Furthermore, determining whether EOCC represents a distinct population warrants a deeper exploration of tumor biology through genetic and molecular profiles.

## Author Contributions

X.C. conducted data analysis. T.X. drafted the manuscript. S.W. downloaded and organized patient data. J.W. contributed to the conception and design of the study. All authors were responsible for and approved the final manuscript.

## Funding

This work was supported by the Natural Science Foundation of Zhejiang Province, LZ23H030001.

## Ethics Statement

Approval of the research protocol by an Institutional Review Board: N/A.

## Consent

The authors have nothing to report.

## Conflicts of Interest

All authors had full access to all of the data in the study and had final responsibility for the decision to submit for publication. The authors declare no conflicts of interest.

## Supporting information


**Table S1:** Baseline Characteristics of Colon Cancer Patients Stratified by Age of Onset.
**Table S2:** Baseline Characteristics of Colon Cancer Patients Stratified by Histology.
**Table S3:** Multivariable Cox Regression Analysis Results for CSS and SSS in EOCC and LOCC Patients.
**Table S4:** Multivariable Cox Regression Analysis Results for CSS and SSS in Colon Cancer Patients Stratified by Age and Histology (EOAC, LOAC, EOMC, LOMC).
**Table S5:** Multivariable Cox Regression Analysis Results for CSS and SSS in Nonmetastatic Colon Cancer Patients.
**Table S6:** Multivariable Cox Regression Analysis Results for CSS and SSS in Metastatic Colon Cancer Patients.

## Data Availability

The data that support the findings of this study are openly available in SEER database at https://seer.cancer.gov/data‐software/, reference number 9.
