# Supplementary material for: Characteristics and Prognosis of Early‐Onset vs. Late‐Onset Colon Cancer: A Propensity Score Matching Analysis Based on Histology
Source: Cancer Med. 2026 Mar 3;15(3):e71681. doi: 10.1002/cam4.71681 (PMC12956548; doi:10.1002/cam4.71681)
Supplement: Supplementary file 1 — Table S1: Baseline Characteristics of Colon Cancer Patients Stratified by Age of Onset. Table S2: Baseline Characteristics of Colon Cancer Patients Stratified by Histology. Table S3: Multivariable Cox Regression Analysis Results for CSS and SSS in EOCC and LOCC Patients. Table S4: Multivariable Cox Regression Analysis Results for CSS and SSS in Colon Cancer Patients Stratified by Age and Histology (EOAC, LOAC, EOMC, LOMC). Table S5: Multivariable Cox Regression Analysis Results for CSS and SSS in Nonmetastatic Colon Cancer Patients. Table S6: Multivariable Cox Regression Analysis Results for CSS and SSS in Metastatic Colon Cancer Patients. [file CAM4-15-e71681-s001.docx]

**Supplementary**

Table S1 Baseline Characteristics of Colon Cancer Patients Stratified by Age of Onset

| **Characteristics** | **Early-onset** | **Late-onset** | **Overall** | **P** |
| --- | --- | --- | --- | --- |
|  | **(N=17402)** | **(N=163402)** | **(N=180804)** |  |
| **Sex** |  |  |  |  |
| Female | 8399 (48.3%) | 81427 (49.8%) | 89826 (49.7%) | <0.001 |
| Male | 9003 (51.7%) | 81975 (50.2%) | 90978 (50.3%) |  |
| **Race** |  |  |  |  |
| Black | 2707 (15.6%) | 19379 (11.9%) | 22086 (12.2%) | <0.001 |
| Others | 2094 (12.0%) | 15249 (9.3%) | 17343 (9.6%) |  |
| White | 12389 (71.2%) | 127564 (78.1%) | 139953 (77.4%) |  |
| Missing | 212 (1.2%) | 1210 (0.7%) | 1422 (0.8%) |  |
| **Marital Status** |  |  |  |  |
| Married | 9329 (53.6%) | 80772 (49.4%) | 90101 (49.8%) | <0.001 |
| Single | 7148 (41.1%) | 72520 (44.4%) | 79668 (44.1%) |  |
| Missing | 925 (5.3%) | 10110 (6.2%) | 11035 (6.1%) |  |
| **Primary Location** |  |  |  |  |
| left | 9436 (54.2%) | 58300 (35.7%) | 67736 (37.5%) | <0.001 |
| right | 7216 (41.5%) | 97313 (59.6%) | 104529 (57.8%) |  |
| Missing | 750 (4.3%) | 7789 (4.8%) | 8539 (4.7%) |  |
| **Differentiation** |  |  |  |  |
| poor differentiation | 2832 (16.3%) | 26775 (16.4%) | 29607 (16.4%) | 0.999 |
| well differentiation | 11982 (68.9%) | 113156 (69.3%) | 125138 (69.2%) |  |
| Missing | 2588 (14.9%) | 23471 (14.4%) | 26059 (14.4%) |  |
| **Stage** |  |  |  |  |
| I | 1716 (9.9%) | 28728 (17.6%) | 30444 (16.8%) | <0.001 |
| II | 4060 (23.3%) | 46336 (28.4%) | 50396 (27.9%) |  |
| III | 5522 (31.7%) | 43225 (26.5%) | 48747 (27.0%) |  |
| IV | 5440 (31.3%) | 35192 (21.5%) | 40632 (22.5%) |  |
| Missing | 664 (3.8%) | 9921 (6.1%) | 10585 (5.9%) |  |
| **T** |  |  |  |  |
| T1 | 1316 (7.6%) | 17737 (10.9%) | 19053 (10.5%) | <0.001 |
| T2 | 1372 (7.9%) | 18766 (11.5%) | 20138 (11.1%) |  |
| T3 | 8428 (48.4%) | 75336 (46.1%) | 83764 (46.3%) |  |
| T4 | 4014 (23.1%) | 29392 (18.0%) | 33406 (18.5%) |  |
| Missing | 2272 (13.1%) | 22171 (13.6%) | 24443 (13.5%) |  |
| **N** |  |  |  |  |
| N0 | 7463 (42.9%) | 90767 (55.5%) | 98230 (54.3%) | <0.001 |
| N1 | 4995 (28.7%) | 38560 (23.6%) | 43555 (24.1%) |  |
| N2 | 3610 (20.7%) | 21829 (13.4%) | 25439 (14.1%) |  |
| Missing | 1334 (7.7%) | 12246 (7.5%) | 13580 (7.5%) |  |
| **M** |  |  |  |  |
| M0 | 11875 (68.2%) | 127435 (78.0%) | 139310 (77.1%) | <0.001 |
| M1 | 5457 (31.4%) | 35286 (21.6%) | 40743 (22.5%) |  |
| Missing | 70 (0.4%) | 681 (0.4%) | 751 (0.4%) |  |
| **Surgery** |  |  |  |  |
| No surgery | 2553 (14.7%) | 25408 (15.5%) | 27961 (15.5%) | 0.00887 |
| Surgery | 14771 (84.9%) | 137183 (84.0%) | 151954 (84.0%) |  |
| Missing | 78 (0.4%) | 811 (0.5%) | 889 (0.5%) |  |
| **Surgery/Radiation** |  |  |  |  |
| No radiation and/or cancer-directed surgery | 16970 (97.5%) | 161396 (98.8%) | 178366 (98.7%) | <0.001 |
| Radiation | 432 (2.5%) | 2006 (1.2%) | 2438 (1.3%) |  |
| **Surgery/Systemic Therapy** |  |  |  |  |
| Surgery | 8077 (46.4%) | 117901 (72.2%) | 125978 (69.7%) | <0.001 |
| Systemic Therapy | 9315 (53.5%) | 45438 (27.8%) | 54753 (30.3%) |  |
| Missing | 10 (0.1%) | 63 (0.0%) | 73 (0.0%) |  |
| **Median household income** |  |  |  |  |
| < $55000 | 2838 (16.3%) | 29950 (18.3%) | 32788 (18.1%) | <0.001 |
| < $65000 | 3053 (17.5%) | 30116 (18.4%) | 33169 (18.3%) |  |
| < $75000 | 4174 (24.0%) | 37908 (23.2%) | 42082 (23.2%) |  |
| >$75000 | 7337 (42.2%) | 65423 (40.1%) | 72760 (40.1%) |  |
| Missing | 0 (0%) | 5 (0.0%) | 5 (0.0%) |  |
| **Rural-urban distribution** |  |  |  |  |
| metropolitan areas | 10421 (59.9%) | 93871 (57.4%) | 104292 (57.7%) | <0.001 |
| intermediate areas | 5055 (29.0%) | 46861 (28.7%) | 51916 (28.7%) |  |
| nonmetropolitan areas | 1873 (10.8%) | 22326 (13.7%) | 24199 (13.4%) |  |
| Missing | 53 (0.3%) | 344 (0.2%) | 397 (0.2%) |  |

Table S2 Baseline Characteristics of Colon Cancer Patients Stratified by Histology

| **Characteristics** | **Adenocarcinoma** | **Mucinous Adenocarcinoma** | **Overall** | **P** |
| --- | --- | --- | --- | --- |
|  | **(N=165085)** | **(N=15719)** | **(N=180804)** |  |
| **Sex** |  |  |  |  |
| Female | 81692 (49.5%) | 8134 (51.7%) | 89826 (49.7%) | <0.001 |
| Male | 83393 (50.5%) | 7585 (48.3%) | 90978 (50.3%) |  |
| **Race** |  |  |  |  |
| Black | 20392 (12.4%) | 1694 (10.8%) | 22086 (12.2%) | <0.001 |
| Others | 16196 (9.8%) | 1147 (7.3%) | 17343 (9.6%) |  |
| White | 127148 (77.0%) | 12805 (81.5%) | 139953 (77.4%) |  |
| Missing | 1349 (0.8%) | 73 (0.5%) | 1422 (0.8%) |  |
| **Marital Status** |  |  |  |  |
| Married | 82401 (49.9%) | 7700 (49.0%) | 90101 (49.8%) | 0.00164 |
| Single | 72467 (43.9%) | 7201 (45.8%) | 79668 (44.1%) |  |
| Missing | 10217 (6.2%) | 818 (5.2%) | 11035 (6.1%) |  |
| **Primary Location** |  |  |  |  |
| left | 63878 (38.7%) | 3858 (24.5%) | 67736 (37.5%) | <0.001 |
| right | 93518 (56.6%) | 11011 (70.0%) | 104529 (57.8%) |  |
| Missing | 7689 (4.7%) | 850 (5.4%) | 8539 (4.7%) |  |
| **Differentiation** |  |  |  |  |
| poor differentiation | 26616 (16.1%) | 2991 (19.0%) | 29607 (16.4%) | <0.001 |
| well differentiation | 114055 (69.1%) | 11083 (70.5%) | 125138 (69.2%) |  |
| Missing | 24414 (14.8%) | 1645 (10.5%) | 26059 (14.4%) |  |
| **Stage** |  |  |  |  |
| I | 28591 (17.3%) | 1853 (11.8%) | 30444 (16.8%) | <0.001 |
| II | 44829 (27.2%) | 5567 (35.4%) | 50396 (27.9%) |  |
| III | 43932 (26.6%) | 4815 (30.6%) | 48747 (27.0%) |  |
| IV | 37474 (22.7%) | 3158 (20.1%) | 40632 (22.5%) |  |
| Missing | 10259 (6.2%) | 326 (2.1%) | 10585 (5.9%) |  |
| **T** |  |  |  |  |
| T1 | 18337 (11.1%) | 716 (4.6%) | 19053 (10.5%) | <0.001 |
| T2 | 18451 (11.2%) | 1687 (10.7%) | 20138 (11.1%) |  |
| T3 | 75657 (45.8%) | 8107 (51.6%) | 83764 (46.3%) |  |
| T4 | 29245 (17.7%) | 4161 (26.5%) | 33406 (18.5%) |  |
| Missing | 23395 (14.2%) | 1048 (6.7%) | 24443 (13.5%) |  |
| **N** |  |  |  |  |
| N0 | 89876 (54.4%) | 8354 (53.1%) | 98230 (54.3%) | <0.001 |
| N1 | 39671 (24.0%) | 3884 (24.7%) | 43555 (24.1%) |  |
| N2 | 22566 (13.7%) | 2873 (18.3%) | 25439 (14.1%) |  |
| Missing | 12972 (7.9%) | 608 (3.9%) | 13580 (7.5%) |  |
| **M** |  |  |  |  |
| M0 | 126800 (76.8%) | 12510 (79.6%) | 139310 (77.1%) | <0.001 |
| M1 | 37584 (22.8%) | 3159 (20.1%) | 40743 (22.5%) |  |
| Missing | 701 (0.4%) | 50 (0.3%) | 751 (0.4%) |  |
| **Surgery** |  |  |  |  |
| No surgery | 26709 (16.2%) | 1252 (8.0%) | 27961 (15.5%) | <0.001 |
| Surgery | 137520 (83.3%) | 14434 (91.8%) | 151954 (84.0%) |  |
| Missing | 856 (0.5%) | 33 (0.2%) | 889 (0.5%) |  |
| **Surgery/Radiation** |  |  |  |  |
| No radiation and/or cancer-directed surgery | 162860 (98.7%) | 15506 (98.6%) | 178366 (98.7%) | 0.997 |
| Radiation | 2225 (1.3%) | 213 (1.4%) | 2438 (1.3%) |  |
| **Surgery/Systemic Therapy** |  |  |  |  |
| Surgery | 115579 (70.0%) | 10399 (66.2%) | 125978 (69.7%) | <0.001 |
| Systemic Therapy | 49443 (30.0%) | 5310 (33.8%) | 54753 (30.3%) |  |
| Missing | 63 (0.0%) | 10 (0.1%) | 73 (0.0%) |  |
| **Median household income** |  |  |  |  |
| < $55000 | 30116 (18.2%) | 2672 (17.0%) | 32788 (18.1%) | <0.001 |
| < $65000 | 30095 (18.2%) | 3074 (19.6%) | 33169 (18.3%) |  |
| < $75000 | 38116 (23.1%) | 3966 (25.2%) | 42082 (23.3%) |  |
| > $75000 | 66753 (40.5%) | 6007 (38.2%) | 72760 (40.2%) |  |
| Missing | 5 (0.0%) | 0 (0.0%) | 5 (0.0%) |  |
| **Rural-urban distribution** |  |  |  |  |
| metropolitan areas | 94822 (57.4%) | 9470 (60.3%) | 104292 (57.7%) | <0.001 |
| intermediate areas | 47667 (28.9%) | 4249 (27.0%) | 51916 (28.7%) |  |
| nonmetropolitan areas | 22218 (13.5%) | 1981 (12.6%) | 24199 (13.4%) |  |
| Missing | 378 (0.2%) | 19 (0.1%) | 397 (0.2%) |  |

Table S3 Multivariable Cox Regression Analysis Results for CSS and SSS in EOCC and LOCC Patients

| Characteristics | CSS | | | Characteristics | SSS | | |
| --- | --- | --- | --- | --- | --- | --- | --- |
|  | Category/Reference | HR (95% CI) | P |  | Category/Reference | HR (95% CI) | P |
| Group/time ≤24 months | EOCC |  |  | Group | EOCC |  |  |
|  | LOCC | 1.34(1.27-1.42) | < 0.001 |  | LOCC | 1.35(1.21-1.50) | < 0.001 |
| Group/time >24 months | EOCC |  |  | Sex | Female |  |  |
|  | LOCC | 1.16(1.06-1.27) | < 0.001 |  | Male | 1.08(1.00-1.17) | 0.055 |
| Sex | Female |  |  | Race | Black |  |  |
|  | Male | 1.13(1.09-1.16) | < 0.001 |  | White | 0.82(0.74-0.91) | < 0.001 |
| Race | Black |  |  |  | Others | 0.76(0.65-0.89) | < 0.001 |
|  | White | 0.88(0.84-0.92) | < 0.001 | Marital status | Married |  |  |
|  | Others | 0.88(0.82-0.95) | < 0.001 |  | Unmarried | 1.34(1.24-1.46) |  |
| Marital status | Married |  |  |  |  |  |  |
|  | Unmarried | N/A (Stratified) |  |  |  |  |  |
| Primary location | left |  |  | Differentiation | well differentiation |  |  |
|  | right | N/A (Stratified) |  |  | poor differentiation | N/A (Stratified) |  |
| Differentiation | well differentiation |  |  | Median household income | < $55000 |  |  |
|  | poor differentiation | N/A (Stratified) |  |  | < $65000 | 0.98(0.86-1.11) | 0.724 |
| Median household income | < $55000 |  |  |  | < $75000 | 0.83(0.73-0.93) | 0.002 |
|  | < $65000 | 0.98(0.92-1.03) | 0.441 |  | > $75000 | 0.74(0.64-0.84) | < 0.001 |
|  | < $75000 | 0.96(0.90-1.01) | 0.131 | Stage |  | N/A (Stratified) |  |
|  | > $75000 | 0.83(0.78-0.88) | < 0.001 |  |  |  |  |
| Rural-urban distribution | metropolitan areas |  |  |  |  |  |  |
|  | intermediate areas | 1.01(0.98-1.06) | 0.471 |  |  |  |  |
|  | nonmetropolitan areas | 1.04(0.97-1.11) | 0.267 |  |  |  |  |
| Stage |  | N/A (Stratified) |  |  |  |  |  |

Table S4 Multivariable Cox Regression Analysis Results for CSS and SSS in Colon Cancer Patients Stratified by Age and Histology (EOAC, LOAC, EOMC, LOMC)

| Characteristics | CSS | | | Characteristics | SSS | | |
| --- | --- | --- | --- | --- | --- | --- | --- |
|  | Category/Reference | HR (95% CI) | P |  | Category/Reference | HR (95% CI) | P |
| Group time≤24 months | EOAC |  |  | Group | EOAC |  |  |
|  | LOAC | 1.06(0.95-1.20) | 0.288 |  | LOAC | 1.30(1.04-1.62) | 0.02 |
|  | EOMC | 1.09(0.92-1.30) | 0.321 |  | EOMC | 1.21(0.87-1.69) | 0.256 |
|  | LOMC | 1.55(1.39-1.72) | <0.001 |  | LOMC | 1.62(1.31-2.00) | <0.001 |
| Group time>24 months | EOAC |  |  | Race | Black |  |  |
|  | LOAC | 1.08(0.91-1.29) | 0.84 |  | White | 0.94(0.73-1.15) | 0.551 |
|  | EOMC | 1.21(0.92-1.58) | 0.464 |  | Others | 1.11(0.81-1.54) | 0.511 |
|  | LOMC | 1.29(1.09-1.53) | 0.037 | Marital status | Married |  |  |
| Race | Black |  |  |  | Unmarried | 1.39(1.17-1.65) | <0.001 |
|  | White | 0.92(0.84-1.00) | 0.061 |  |  |  |  |
|  | Others | 0.92(0.80-1.06) | 0.265 |  |  |  |  |
| Marital status | Married |  |  | Differentiation | well differentiation |  |  |
|  | Unmarried | 1.32(1.24-1.41) | <0.001 |  | poor differentiation | 1.82(1.51-2.20) | <0.001 |
| Differentiation |  |  |  | Median household income | < $55000 |  |  |
|  |  |  |  |  | < $65000 | 1.11(0.86-1.44) | 0.418 |
| Median household income | < $55000 |  |  |  | < $75000 | 0.87(0.69-1.11) | 0.263 |
|  | < $65000 | 1.00(0.91-1.12) | 0.904 |  | > $75000 | 0.60(0.46-0.77) | <0.001 |
|  | < $75000 | 0.99(0.89-1.09) | 0.766 | Stage |  | N/A (Stratified) |  |
|  | > $75000 | 0.83(0.75-0.92) | <0.001 |  |  |  |  |
| Stage |  | N/A (Stratified) |  |  |  |  |  |

Table S5 Multivariable Cox Regression Analysis Results for CSS and SSS in Nonmetastatic Colon Cancer Patients

| Characteristics | CSS | | | Characteristics | SSS | | |
| --- | --- | --- | --- | --- | --- | --- | --- |
|  | Category/Reference | HR (95% CI) | P |  | Category/Reference | HR (95% CI) | P |
| Group/time ≤24 months | EOAC |  |  | Group | EOAC |  |  |
|  | LOAC | 0.96(0.76-1.21) | 0.719 |  | LOAC | 0.61(0.44-0.84) | 0.003 |
|  | EOMC | 1.34(0.97-1.86) | 0.08 |  | EOMC | 1.16(0.76-1.76) | 0.493 |
|  | LOMC | 2.14(1.75-2.61) | <0.001 |  | LOMC | 1.57(1.21-2.03) | <0.001 |
| Group/time >24 months | EOAC |  |  |  |  |  |  |
|  | LOAC | 0.88(0.65-1.19) | 0.591 |  |  |  |  |
|  | EOMC | 1.29(0.93-1.80) | 0.87 | Race | Black |  |  |
|  | LOMC | 1.23(1.11-1.62) | <0.001 |  | White | 0.82(0.62-1.09) | 0.17 |
| Sex | Female |  |  |  | Others | 0.97(0.61-1.56) | 0.906 |
|  | Male | 1.14(1.02-1.27) | 0.026 | Marital status | Married |  |  |
| Race | Black |  |  |  | Unmarried | 1.38(1.11-1.72) | 0.004 |
|  | White | 0.85(0.73-0.99) | 0.034 | Differentiation | well differentiation |  |  |
|  | Others | 0.94(0.75-1.170 | 0.59 |  | poor differentiation | 2.74(2.15-3.49) | <0.001 |
| Marital status | Married |  |  | Median household income | < $55000 |  |  |
|  | Unmarried | 1.35(1.21-1.50) | <0.001 |  | < $65000 | 0.93(0.67-1.30) | 0.689 |
| Median household income | < $55000 |  |  |  | < $75000 | 0.88(0.65-1.19) | 0.408 |
|  | < $65000 | 0.95(0.80-1.12) | 0.522 |  | > $75000 | 0.57(0.41-0.79) | <0.001 |
|  | < $75000 | 0.94(0.80-1.10) | 0.442 |  |  |  |  |
|  | > $75000 | 0.76(0.65-0.89) | <0.001 |  |  |  |  |
| Differentiation |  | N/A (Stratified) |  |  |  |  |  |

Table S6 Multivariable Cox Regression Analysis Results for CSS and SSS in Metastatic Colon Cancer Patients

| Characteristics | CSS | | | Characteristics | SSS | | |
| --- | --- | --- | --- | --- | --- | --- | --- |
|  | Category/Reference | HR (95% CI) | P |  | Category/Reference | HR (95% CI) | P |
| Group | EOAC |  |  | Marital status | Married |  |  |
|  | LOAC | 1.09(0.98-1.22) | 0.122 |  | Unmarried | 1.36(0.97-1.89) | 0.073 |
|  | EOMC | 1.06(0.89-1.260 | 0.537 | Differentiation | well differentiation |  |  |
|  | LOMC | 1.23(1.09-1.39) | <0.001 |  | poor differentiation | 2.25(1.57-3.23) | <0.001 |
| Race | Black |  |  |  |  |  |  |
|  | White | 0.90(0.79-1.02) | 0.087 |  |  |  |  |
|  | Others | 0.86(0.70-1.05) | 0.143 |  |  |  |  |
| Marital status | Married |  |  |  |  |  |  |
|  | Unmarried | 1.30(1.19-1.43) | <0.001 |  |  |  |  |
| Median household income | < $55000 |  |  |  |  |  |  |
|  | < $65000 | 1.04(0.90-1.20) | 0.576 |  |  |  |  |
|  | < $75000 | 0.99(0.86-1.14) | 0.912 |  |  |  |  |
|  | > $75000 | 0.88(0.76-1.00) | 0.057 |  |  |  |  |
| Primary location |  | N/A (Stratified) |  |  |  |  |  |
| Differentiation |  | N/A (Stratified) |  |  |  |  |  |
